# Palliative care nurses’ views on their retention intention: a qualitative study

**DOI:** 10.3389/frhs.2026.1822219

**Published:** 2026-06-24

**Authors:** Zongwen Yi, Qing Yu, Haoyue Zhao, Ping Huang, Yanjia Li

**Affiliations:** 1 Department of Pediatrics, Sichuan Taikang Hospital, Chengdu, Sichuan, China; 2 Department of Gynaecology and Obstetrics, Sichuan Taikang Hospital, Chengdu, Sichuan, China; 3 Department of General Practice, Sichuan Taikang Hospital, Chengdu, Sichuan, China; 4 Department of Nursing, Sichuan Taikang Hospital, Chengdu, Sichuan, China

**Keywords:** Job Demands-Resources Model, palliative care nurse, retention intention, turnover intention, work requirements

## Abstract

**Background:**

Palliative care is in short supply worldwide, and nurses are core members of its implementation. However, there is currently a severe shortage of palliative care nurses. Therefore, enhancing their retention intention and attracting more nurses to join this field is an urgent issue that needs to be addressed.

**Methods:**

This research is based on the theoretical framework of the Job Demands-Resources Model (JD-R). In the qualitative research, the phenomenological research method was adopted,And a semi-structured interview guide was used to collect the data. Each interview lasts for approximately 20 to 52 min. The data is analyzed using the thematic analysis method.

**Results:**

This study involved 15 participants. In the area of retention intention, we have identified an excessively high level of job demands (continuous exposure to death and near-death experiences, High-intensity emotional labor, complex communication and ethical dilemmas, as well as intense physical labor) are the obstructive factors affecting retention intention.However, sufficient work resources (team support and cohesion, organizational support and recognition, sense of job value, and a humanized and supportive environment) and personal resources (sense of mission and altruistic values, psychological resilience and self-adjustment, reconstruction of the understanding of life and death, professional skill) are important factors in promoting the intention to remain in the job.

**Conclusion:**

This study reveals that the Palliative care nurse retention intention in their positions is a complex phenomenon influenced by multiple factors. It emphasizes that managers should recognize the value of palliative care nurses’ work, reduce their workload demands, optimize their job content, provide resources that can empower and motivate the nurses, and attach importance to the enhancement of the nurses’ intrinsic personal resources.

## 1 Introduction

The world is currently undergoing an unprecedented demographic shift, with the proportion of the elderly population increasing significantly (1). This trend of population aging has directly led to a sharp increase in the demand for end-of-life care related to aging and chronic diseases (such as cancer, cardiovascular diseases, and neurodegenerative disorders) (2).

An update of The Lancet Commission on global access of palliative care and pain relief, between 1990 and 2021, the global burden of severe related health distress increased by 74%, while there is a significant gap between the demand for and the supply of palliative care, and this gap is expected to expand significantly in the coming decades (3). By 2060, the demand for hospice care (measured by the prevalence of severe health or disease-related pain) is projected to increase by 87% (4). However, only 14% of the global demand for hospice care is being met (5), The phenomenon that the demand for Palliative Care in middle- and low-income countries remains unmet is particularly evident (6). Palliative Care, as a multidisciplinary approach focused on improving the quality of life for patients facing life-threatening conditions and their families, centers around the prevention and alleviation of suffering, providing pain and symptom management, as well as psychological, social, and spiritual support (7).

In the face of the arrival of an aging global society and the imbalance in the supply and demand of palliative Care, all countries are actively responding. In China, from 2017 to 2023, 185 national-level pilot hospice care wards were established in three batches (with an additional 61 cities/districts including Beijing and Zhejiang in 2023). By 2025, it is planned to achieve the goal of having at least one hospice ward in each county and hospice beds in all grassroots institutions. In 2025, Beijing will introduce the country's first local standard, “Norms for Hospice Care Services in Medical Institutions”, and other measures to promote the development of hospice care.

Nursing staff in palliative care are the main core members responsible for implementing palliative care. They are at the forefront of the care process, undertaking crucial duties such as complex clinical assessments, symptom management, medication adjustments, patient and family education, emotional support, communication coordination, and end-of-life care (8, 9). Studies have shown that nurse-led supportive care can improve the quality of life of cancer patients, maintain their role function domains, and maintain the activity and independence of patients with advanced cancer (10, 11).

However, the shortage of nurses is a common, widespread and persistent problem (12). Nursing staff in palliative care frequently encounter death, heavy workloads, and complex interpersonal relationships, which can easily lead to occupational burnout (13) and compassion fatigue (14), Compared with nurses in other departments, they face more difficulties and pressures, and most nurses are unwilling to engage in palliative care work (15–17).

The intention of palliative care nurses to remain in their retention to the subjective willingness of registered nurses working in the field of palliative care to stay in their jobs for a long time (18). This concept emphasizes an autonomous and proactive psychological state and behavioral tendency. It is not only the most direct and effective key antecedent variable for predicting turnover behavior, but also an important indicator for measuring the stability of palliative care teams, the quality of care, as well as nurses’ own professional identity and sense of happiness.

The current research on nurses’ retention intentions shows the following characteristics: The research focus is asymmetric, A large number of studies focus on the intention to leave and its core antecedents - job burnout and compassion fatigue, rather than the positive retention intention (19–21). The focus of the research method is on quantitative research (such as questionnaire surveys), which dominates (22). The positive factors related to retention are often hidden within qualitative studies on “career resilience”, “meaning construction”, “work experience”, “support needs”, and it is necessary to extract the reasons that motivate them to stay from the accounts of the respondents. To fill the aforementioned research gap, this study will employ qualitative research methods to systematically explore the key driving factors and obstacles that influence the retention intention of hospice care nurses. The results of this study not only have significant practical significance for formulating effective management strategies for nursing staff, improving the quality of palliative care services, and meeting the demands of an increasingly aging population, but also provide new perspectives and support for related theoretical research.

## 2 Methods

### 2.1 Design

Choose the phenomenological research method in qualitative research, adopt semi-structured interviews to collect data. According to the research purpose, consult relevant literature, discuss with experts to formulate the interview outline, and through a pre-interview with two people, determine the final outline.

### 2.2 Guiding framework

The theoretical foundation of this study is based on the established Job Demands-Resources Model (JD-R) (23).

This model is a comprehensive and flexible heuristic framework, which assumes that all occupational characteristics can be divided into two categories: job demands and job resources. Excessive work demands (those that require continuous physical or mental effort, accompanied by physical and psychological costs) can lead to energy depletion, subsequently causing health problems. This path is called the path of impaired health. In the field of nursing, long-term exposure to high work demands (such as emotional needs and time pressure) is the main cause of occupational burnout (characterized by emotional exhaustion and reduced personal sense of achievement). And job burnout, in turn, is recognized as a common predictor of adverse consequences (such as decreased work performance, deteriorating health conditions, and increased likelihood of leaving the job) (24). Sufficient working resources (those that contribute to achieving work goals, reducing work demands, and stimulating personal growth and development) can foster an active and motivated working state (25), This path is called the incentive path.

Work resources can exist at the organizational level (such as superior support, participation in decision-making), at the interpersonal level (such as team cohesion, social support), or at the task level (such as skill diversity, work autonomy). These resources can enhance work engagement, thereby increasing organizational commitment and job satisfaction, and subsequently strengthening the intention to stay in the organization. The JD-R model also acknowledges that there may be interactions between these two processes.

**Figure 1:**
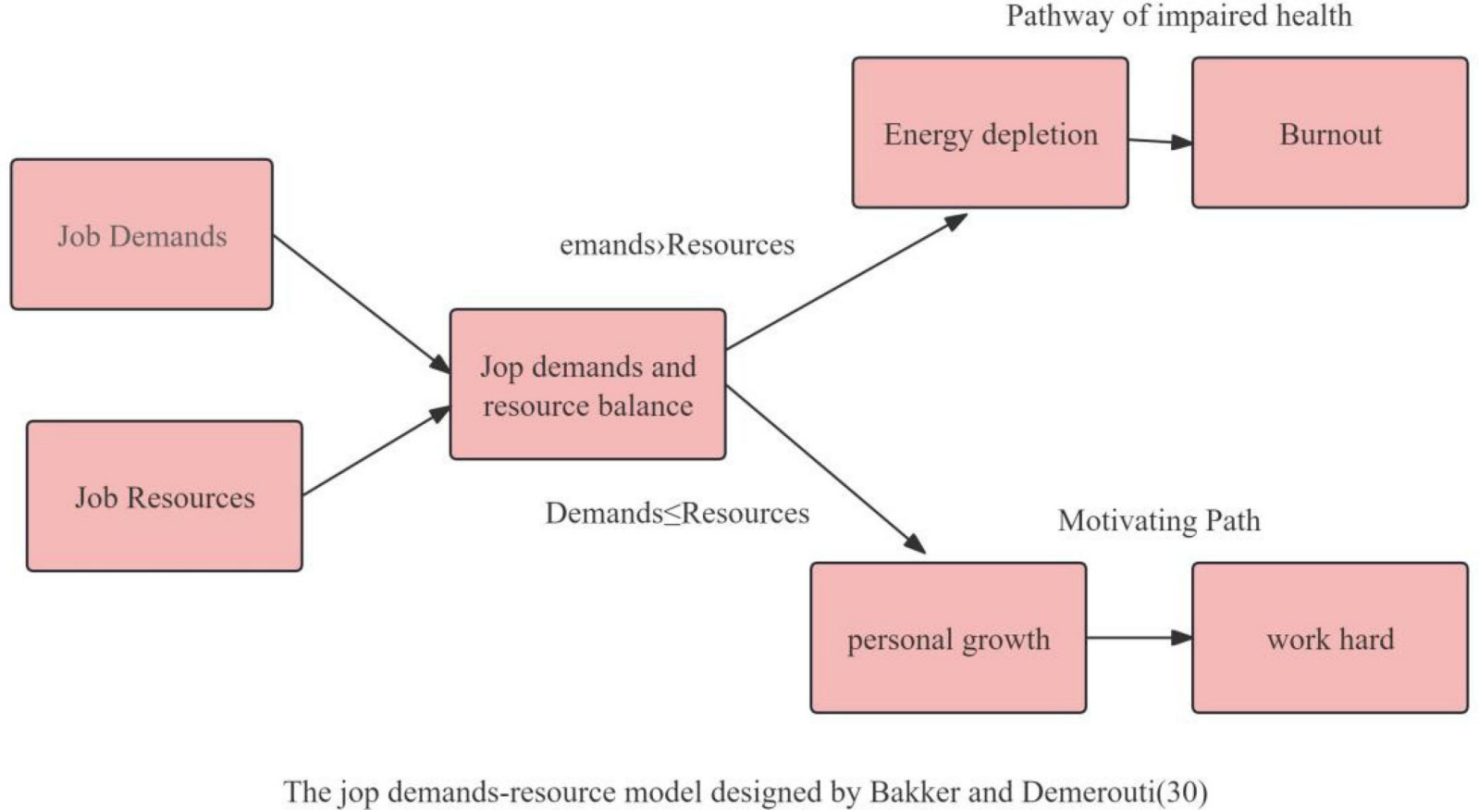
The jop demands-resource model designed by bakker and demerouti.

Work resources can buffer the negative impact of work demands on job burnout. For instance, strong support from colleagues or a positive team atmosphere can mitigate the negative impact of high emotional demands on the well-being of nurses (26). This model is particularly suitable for studying job burnout and retention intention in high-pressure occupations such as palliative care, as well as the impact of work resources on nurse retention or turnover (27, 28). Provides an overview of this model Figure 1 (29).

### 2.3 Participant

We recruited nurses engaged in palliative care from a tertiary general teaching hospital in Sichuan, China. The hospital has 1,001 beds and is a private medical institution. Using purposive sampling, the inclusion criteria were: registered nurses who had been working in the hospice care department for at least 12 months; the exclusion criteria were: nurses on long-term leave and those who refused to participate in this interview; diversity guarantee: we intentionally included nurses of different ages (22–43 years old), different working years (1–20 years), and different ranks (nursing assistants - senior nurses). These nurses include both junior nurses and intermediate nurses, as well as senior nurses, representing nurses at all stages of the palliative care profession. We had already made contact and received support from the head nurse by phone. The head nurse gave a brief update at the nurse team meeting. After the initial notification, we conducted one-on-one conversations and made phone calls to the nurses to explain the research and answer their questions. No nurse was pressured or asked to report their decisions to the head nurse. Obtain the informed consent for the nurse interviews and make appointments for the time. During the formal interviews, obtain the written informed consent from the nurses. To protect the information of the interviewees, we assigned codes to them. Participant characteristics are presented in Table 1.

**Table 1:** Characteristics of nurses involved in palliative care.

Number	years	Gender	Education level	technical title	Working experience (years)	marital status
L1	43	Femininity	Bachelor degree	Co-chief superintendent nurse	10–20 years	Married
L2	35	Femininity	Bachelor degree	Supervisor nurse	10–20 years	Married
L3	30	Femininity	Bachelor degree	Supervisor nurse	4–10 years	Spinsterhood
L4	29	Femininity	Bachelor degree	Supervisor nurse	4–10 years	Spinsterhood
L5	28	Femininity	Bachelor degree	Supervisor nurse	4–10 years	Spinsterhood
L6	38	Femininity	Bachelor degree	Supervisor nurse	10–20 years	Married
L7	23	Femininity	Bachelor degree	Junior nurse	1–3 years	Spinsterhood
L8	39	Femininity	Bachelor degree	Supervisor nurse	10–20 years	Married
L9	22	Femininity	Associate degree	Junior nurse	1–3 years	Spinsterhood
L10	29	Femininity	Bachelor degree	Supervisor nurse	4–10 years	Spinsterhood
L11	42	Femininity	Bachelor degree	Supervisor nurse	10–20 years	Married
L12	32	Femininity	Bachelor degree	Supervisor nurse	10–20 years	Spinsterhood
L13	31	Femininity	Bachelor degree	Supervisor nurse	10–20 years	Married
L14	27	Masculinity	Bachelor degree	Supervisor nurse	4–10 years	Married
L15	32	Femininity	Bachelor degree	Supervisor nurse	10–20 years	Spinsterhood

### 2.4 Data collection

We conducted the interviews using a semi-structured approach. The interview was conducted by two researchers from September 1st to October 20th, 2024. Before the interview, the participants were informed of the purpose of the study and the confidentiality of the interview content. It was also explained that the interview would be recorded and they were asked to provide written consent. The members of the research team and the interviewees came from the same hospital but worked in different departments. The members of the research team had never held any management positions. During the interviews, there was no head nurse present. The participants were assured of anonymity and were informed that there would be no consequences for not participating.

During the interview, one interviewer followed the outline to conduct the interview, asking open-ended questions to the interviewee. They used phrases like ‘Could you elaborate further? Please tell me more’ to encourage the interviewee. The other researcher mainly recorded the interviewee's expressions and changes in their activities. We invited 19 nurses specializing in palliative care. Among them, 15 accepted the invitation and participated in the interview, while 4 declined our request for an interview.

The participants in the interview included 2 individuals who conducted the online interview via Tencent Meeting. 13 people participated in the face-to-face interview. The interview duration ranged from 20 to 52 min, with an average of 31 min. Data collection continued until the research team judged analytic sufficiency; saturation was not formally tested. The decision to stop at 15 participants was made when the team agreed that no new practical insights emerged across the dimensions of job demands and job resources. According to the research by Hennink & Kaiser, in a relatively homogeneous population and when focusing on a specific research question, a sample size of 9 to 17 individual interviews is typically sufficient to reach saturation, which supports the adequacy of our sample size (30).

### 2.5 Data analysis

Data analysis was conducted using the Thematic Analysis method. After the interview, two researchers separately compiled the verbatim transcripts of the interview within 24 h. They read the transcripts repeatedly, took notes of their initial thoughts while reading, marked potential key statements (such as the patient's original words, contradictory expressions) with different colors, and wrote a memo to summarize the overall impression, tag each sentence and paragraph, generate the initial code, organize it using NVivo, create the main topics and sub-topics, and have the two researchers cross-check each other's work. Mark the controversial areas, and the research team holds regular reporting meetings (every 3–4 interviews later) to discuss how the background of each researcher might affect data collection and interpretation. Through discussions, they resolve differences and ultimately determine the topic and generate the report. Finally, determine the main topics and generate the final report.

### 2.6 Approvals and ethical considerations

This research was approved by the Ethics Committee of Sichuan Taikang Hospital. (SCTKIRB−2024−014). The interviewers’ data are all encoded for identification. Before the interview, the interviewees’ informed consent was obtained. The interviewees can withdraw from the study at any time. Additionally, we guarantee that the personal information of every participant remains confidential, and the interview results are solely utilized for statistical analysis.

### 2.7 Precision

In order to enhance the rigor, credibility and applicability of the research, we employed the 6-step thematic analysis method proposed by Braun and Clarke in the data analysis process. This is a well-known qualitative approach (31). At the same time, this research was guided by the Job Demands-Resources Model, JD-R framework (23), in addition, a large number of transcriptions, field notes, memos and recordings were also employed to further enhance the credibility of the research.

### 2.8 Patient and public participation statement

It was not designed, implemented, reported or disseminated by patients or the public. Patients or the public were not involved because this study focused specifically on the perspectives of healthcare professionals, aiming to identify the facilitating and obstructive factors influencing the retention of hospice nurses from the provider's perspective.

## 3 Results

After interviewing 15 hospice care nurses, we identified 4 main themes, each of which has 4 sub-themes. The main topic and core issues are presented in Table 2.

**Table 2:** Topic and core issues discussed.

Primary Topic	Secondary subtopic	Core interview questions	Core Answer
Job Demands Leading to Emotional Exhaustion and Turnover Intention)	Continuous exposure to death and near-death experiences	Frequently witnessing the death of patients, how did you adapt and view this process?	Sometimes when I see patients die, I become numb. I feel like I'm a machine without emotions.
	High-intensity emotional labor	When faced with the sorrowful emotions of patients and their families, how do you usually handle the situation? Do you need to adjust or hide your true emotions?	Every time I see a child die, I think of my own child. Just like the parents of that child, I can't get over it.
	Complex communication and ethical dilemmas	When communicating the patient's condition or making end-of-life decisions with the family members, what difficulties or dilemmas have you encountered?	Facing the helplessness of the family members, I really wanted to comfort them, but I didn't know how to start.
	Intense physical labor	Does the work you do at your job make you feel tired?	Each day, as the workday draws to a close, exhaustion envelops me, rendering speech and movement arduous tasks
Job Resources that Empower and Motivate Retention	Team support and cohesion	How do you evaluate your team?	Our current working atmosphere is very good, and this is the main reason why I decided to stay on
	Organizational support and recognition	What kind of support do you think the hospital or department management has provided for you?	Currently, two of us work the night shift together. This is wonderful and it's also a key reason for me to stay
	Sense of job value	Despite the challenges of the job, what makes you feel that this job is valuable and meaningful?	In that instant, I truly felt like one of their own, sensing their deep approval
	Humanized and supportive environment	Which parts of the hospital moved you the most?	The current working environment is truly excellent. It makes people feel very calm.
The Central Role of Personal Resources in the Dynamic Balance between Demands and Resources	Sense of Mission and Altruistic Values	How do you understand the role of a hospice nurse?	Assisting a person in having a peaceful farewell and a dignified exit from this world is an exceptionally great act of kindness
	Psychological Resilience and Self-Adjustment Strategies	What methods do you use to relieve the stress and mental burden caused by work?	I participated in the “Life Dialogue” event organized by the hospital. Every time I finished attending it, I felt my energy returning
	Reconstruction of the perception of life and death	What growth or changes has this work experience brought about for you personally?	Working in palliative care has taught me to release the past, to concentrate on the present moment, and to treasure every instan
	professional skill	Can you handle the job of Palliative care?	I came across a 10-year-old boy who wouldn't cooperate with any treatment. So I adopted the method of narrative medicine, gradually helping the child lower his guard and accept the treatment.

### 3.1 Job demands leading to emotional exhaustion and turnover intention

Work demands refer to the objective physical, psychological, social or organizational needs that exist in the workplace, which require continuous physical or mental effort to accomplish tasks and fulfill specific criteria (32). This study reveals that hospice nurses are confronted with a variety of unique and highly demanding work requirements, which continuously deplete their emotional and psychological resources and are the primary reasons for their emotional exhaustion and the emergence of the intention to leave their jobs. This topic perfectly validates the “health loss process” in the JD-R model. Through the analysis of the interview data, we further refined these into four sub-themes: continuous exposure to death and near-death experiences, high-intensity emotional labor, complex communication and ethical dilemmas, as well as intense physical labor.

#### 3.1.1 Continuous exposure to death and near-death experiences

Most nurses report that working in the palliative care department is more stressful than in other departments (L3, L6, L8, L11, L12, L15).

Over the past few days, we've had patients die in the hospital but there's no place to store their bodies. We had to leave them in the hospital. This has been a significant shock for us (L16).

Sometimes when I see patients die, I become numb. I feel like I'm a machine without emotions (L1).

Sometimes I think life is really boring and meaningless. It's so empty. Eventually, we all have to die. I really hate this feeling (L9).

#### 3.1.2 High-intensity emotional labor

High-intensity emotional labor is a psychological issue that is commonly mentioned by nurses (L2, L5, L8, L10, L11, L15). Palliative care is a practice that encompasses multiple aspects such as spirituality, psychology and physical health. Nurses find it difficult to distinguish between professional relationships and personal relationships within this context. When faced with the death of a patient, they often experience severe emotional issues just like the family members (33, 34). This process resulted in a significant depletion of inner energy.

Recently, a patient who was very close to me passed away. I was extremely sad. I regarded her as a family member and even dreamed about her in my sleep (L3).

Every day, one has to deal with desperate family members and patients. One has to find various ways to offer them help and comfort. It's very exhausting every day (L9).

More than half of the nurses indicated that providing palliative care for children presented greater emotional challenges for them（L1, L3, L5, L6, L8, L9, L11, L13).

If we are to continue working with children with autism in the future, I think I won't be able to keep doing this job for a long time (Crying) (L11).

Every time I see a child die, I think of my own child. Just like the parents of that child, I can't get over it (L13).

#### 3.1.3 Complex communication and ethical issues

Nurses are tasked with the daunting responsibility of communicating intricate information while offering emotional solace amidst intense pressure. They frequently grapple with finding equilibrium between “exerting every effort to save” and “permitting the patient to depart peacefully”. This cognitive conflict is a significant source of stress.

Some of the family members of the terminally ill patients strongly requested for emergency treatment. Seeing the patient unable to leave this world peacefully made me feel that I was not qualified to be a competent palliative care nurse（L3).

Facing the helplessness of the family members, I really wanted to comfort them, but I didn't know how to start (L6).

I am currently managing a patient with advanced cancer who remains silent every day. I have no idea how to break this deadlock (L8).

At times, we are confronted with the reality of death, yet remain uncertain about how to proceed (L11).

#### 3.1.4 Intense physical labor

Apart from the emotional and cognitive strain, the heavy physical demands also pose a major challenge in the daily work of hospice nurses. Due to the patient's complete loss of self-care ability, all the basic care, position changes, and hygiene cleaning tasks need to be carried out personally by the nurses. This places a continuous burden on the nurses’ bodies and often intertwines with emotional labor, intensifying the overall sense of exhaustion.

Among some of the patients’ families, although they fully understand that there is little hope for their loved one’s recovery, they still plead with us to continue the treatment at any cost (L4).

Within our department, a multitude of treatments await execution. We are so engrossed in administering these diverse therapies that we scarcely find the time to discern the true needs of our patients. This, I believe, is a misstep (L8).

Patients undergoing palliative care, in truth, yearn for our companionship more profoundly, yet we are perpetually short of time (L10).

I aspire to offer more comprehensive life care and I strive to provide psychological care for the patients, yet the sheer volume of treatments renders it impossible to complete them all each day. Time, a scarce commodity, leaves me unable to attend to these tasks, filling me with frustration and regret (L15).

Each day, as the workday draws to a close, exhaustion envelops me, rendering speech and movement arduous tasks (L16).

### 3.2 Job resources that empower and motivate retention

Although the work was challenging, this study also identified a large number of key resources that empowered nurses and motivated their retention. These resources effectively alleviated the adverse effects of work demands, fulfilled the fundamental psychological needs of nurses, enhanced their work engagement and dedication, and clearly illustrated the “Motivating Path” in the JD-R model. These resources are mainly reflected in four sub-themes: team support and cohesion, organizational support and recognition, sense of job value, and a humanized and supportive environment.

#### 3.2.1 Team support and cohesion

A multidisciplinary team with a shared vision and providing seamless support is the most highly valued social work resource. The deep understanding, emotional resonance and professional complementarity among team members have created a safe and inclusive environment, which serves as the most important stress buffer and emotional refueling station for nurses.

Our current working atmosphere is very good, and this is the main reason why I decided to stay on (L1).

The working atmosphere is even more important to me than other benefits (L2).

Among us nurses, we can identify each other's shortcomings and help each other at work. We also get along well with the doctors, who understand the difficulties of being nurses. The head nurse is also very caring towards us, providing us with sufficient rest during the shift arrangement. I think this is very important (L3).

It’s hard for me to imagine that if colleagues don't get along well with each other, going to work would definitely be a very painful experience (L12).

#### 3.2.2 Organizational support and recognition

Substantial organizational support from the hospital level is the foundation for nurses to feel valued and protected. This includes fair compensation, adequate staffing, systematic training, and the public recognition by leaders of the value of their work. These resources directly meet the survival, safety and development needs of nurses.

Most nurses stated that their current workload is as busy as that of ICU nurses, but they receive the same remuneration as those in general wards. This is unfair to them, and they hope that hospital leaders can have a better understanding of palliative care nurses and provide them with corresponding salaries (L2, L5, L7, L8, L10, L11).

If we could provide us with a more reasonable allocation of personnel, I would be able to work in the field of palliative care for a longer period of time（L6).

Currently, two of us work the night shift together. This is wonderful and it's also a key reason for me to stay (L12).

I heard that we will be required to work the night shift alone in the future. That's really terrifying (L13).

I haven't received any training related to death. When I see a patient die now, I feel scared. I hope there are senior nurses with me to face death together (L15).

#### 3.2.3 The sense of value in work

The profound sense of value derived from “guarding a good ending” in one's work is the most powerful internal motivating resource. This sense of achievement and fulfillment derived from the work itself cannot be replaced by external rewards. It directly strengthens the nurses’ professional identity and retention determination.

One of the elderly people I cared for passed away. When his family came to collect his body, they asked me to help him put on his clothes. His family said, “Because it was during the final moments that I was taking care of him and had a good relationship with him. He must have wanted the clothes he wore at the end to be the ones I helped him prepare” (l5).

At the farewell ceremony As I cared for the patient, their family members each came up to embrace me warmly. In that instant, I truly felt like one of their own, sensing their deep approval. It was an incredibly precious experience (L10).

Following the interview, a nurse shared with me a screenshot from a WeChat chat. It revealed that after one of her patients had passed, the family had brought fruits they had grown themselves to show their heartfelt gratitude. She said, “If you ask me what the value of a hospice care nurse is, I think this is the best answer” (L9)

#### 3.2.4 Humanized and supportive working environment

A well-designed working environment imbued with humanistic care serves as the fundamental resource for ensuring the high-quality execution of palliative care nurses’ duties. It goes beyond the realm of interpersonal interaction, encompassing the comfort of the physical space, the convenience of the facilities, as well as the serenity and dignity of the overall atmosphere. This environment itself is silently supporting the nurses’ work, effectively alleviating their additional burdens and vividly conveying the organization's core value of caring for both its employees and patients.

The current working environment is truly excellent. It has a large space and the decoration is very cozy, which perfectly conveys the feeling of hospice care. It makes people feel very calm.

There is also a duty room. After working the night shift, we can get a good rest in the department (L5).

There are always various snacks provided by doctors and head nurses in our duty room (L9).

The lighting in our department is rather soft (L15).

### 3.3 The central role of personal resources in the dynamic balance between demands and resources

This study has revealed that the intrinsic personal resources of nurses play a pivotal role in sustaining their retention intentions. Personal resources refer to the positive self-evaluations and abilities that individuals possess and which enable them to effectively control their environment and achieve their goals (such as resilience, optimism, and a sense of mission) (35). These intrinsic qualities are not static; rather, they are refined and elevated through interaction with the working environment. They can both directly empower and regulate the relationship between work demands and exhaustion, and are the key variables determining whether nurses can persevere in the face of challenges. This study identified four core sub-themes of personal resources: sense of mission and altruistic values; psychological Resilience and Self-Adjustment; Reconstruction of the Understanding of Life and Death; professional skill.

#### 3.3.1 Sense of mission and altruistic values

An inherent sense of “being called upon” and a core value of helping others are the most fundamental personal resources (36). It acts as a “meaning filter” for work, re-evaluating challenging demands as a means to achieve personal value, thereby providing a continuous internal driving force.

I firmly believe in the principle of cause and effect. Working in palliative care is akin to sowing a seed of kindness. Assisting a person in having a peaceful farewell and a dignified exit from this world is an exceptionally great act of kindness (L3).

When my grandma passed away, she didn't receive proper care. This is a regret of mine. I want to ensure that every patient in my work can leave this world with dignity（L4).

Many years ago, I made a firm decision to continue doing this job (L8).

With our assistance, the patients and their families are gradually coming to accept death in a proper manner, and seeing that they no longer fear it. This is the greatest significance of our work (L10).

#### 3.3.2 Psychological resilience and self-adjustment

Psychological Resilience does not mean being immune to pain, but rather the ability to “bounce back” and recover after facing adversity (37). The nurses manage stress by actively adopting various cognitive and behavioral strategies, protecting their psychological boundaries. This self-regulation ability is a crucial personal resource that can be acquired.

I often find joy in shopping and playing games. These leisure activities serve as effective ways for me to relieve stress (L5).

I mainly relieve stress by playing games (L6).

I participated in the “Life Dialogue” event organized by the hospital. Every time I finished attending it, I felt my energy returning (L9).

#### 3.3.3 Reconstruction of the understanding of life and death

The reconfiguration of the perception of life and death refers to the philosophical understanding of life, Death that is acquired during the hospice care process and is more open-minded and profound. It is a unique and powerful personal resource (38). This cognitive shift can transform the emotional demands at work from a psychological threat into a resource for life education, fundamentally altering the relationship between nurses and their work.

Working in palliative care has taught me to release the past, to concentrate on the present moment, and to treasure every instant (L3).

I've come to understand that death is beyond our control. We must prepare for it beforehand. I intend to draft and finalize my pre-mortem will (L4).

After working in palliative care, I witnessed the fragility and disappearance of life, but also saw the preciousness and brilliance of life, exemplified by love and family affection, hold a significance that, in my view, surpasses life itself (L8).

#### 3.3.4 Professional skill

Outstanding palliative care professional skills are a highly valued personal asset for nurses. These skills extend beyond basic care, encompassing advanced capabilities such as complex symptom management, communication regarding advance care planning, and grief counseling. Mastering these skills has brought about a strong sense of professional self-efficacy for Nurses, that is, they have confidence in their ability to successfully complete a certain task. Having this kind of “I can handle it” confidence is the fundamental inner foundation that enables them to face the huge challenges in their work and gain a sense of achievement from it.

Our hospital has been promoting narrative medicine, and I feel it is particularly beneficial for patients undergoing palliative care (L1).

I came across a 10-year-old boy who wouldn't cooperate with any treatment. So I adopted the method of narrative medicine, gradually helping the child lower his guard and accept the treatment. During that period, we also discussed the topic of death with the child (L7).

Our job requires not only excellent first aid skills but also the ability to treat patients of all ages, from infants to the elderly. The clinical skills needed must be comprehensive. At the same time, one must possess professional skills in palliative care such as narrative medicine and spiritual care. Hence, individuals capable of thriving in this environment are truly exceptional (L10).

## 4 Discussion

This study, through qualitative methods, deeply explored the key factors influencing the retention intention Palliative care nurses, and successfully integrated these factors into the Job Demands-Resources (JD-R) theoretical framework.Our analysis reveals a complex dynamic process: The high-intensity work demands (emotional labor, exposure to death, communication difficulties, physical exhaustion) trigger emotional exhaustion and the intention to leave through the process of health deterioration. While abundant working resources (team support, organizational recognition, humanized environment, and a sense of job value) stimulate work engagement through the motivational process and promote job retention. More importantly, the personal resources of nurses (such as a sense of mission, professional skills, resilience, and wisdom regarding life and death) play a central role in this dynamic balance.

**Figure 2:**
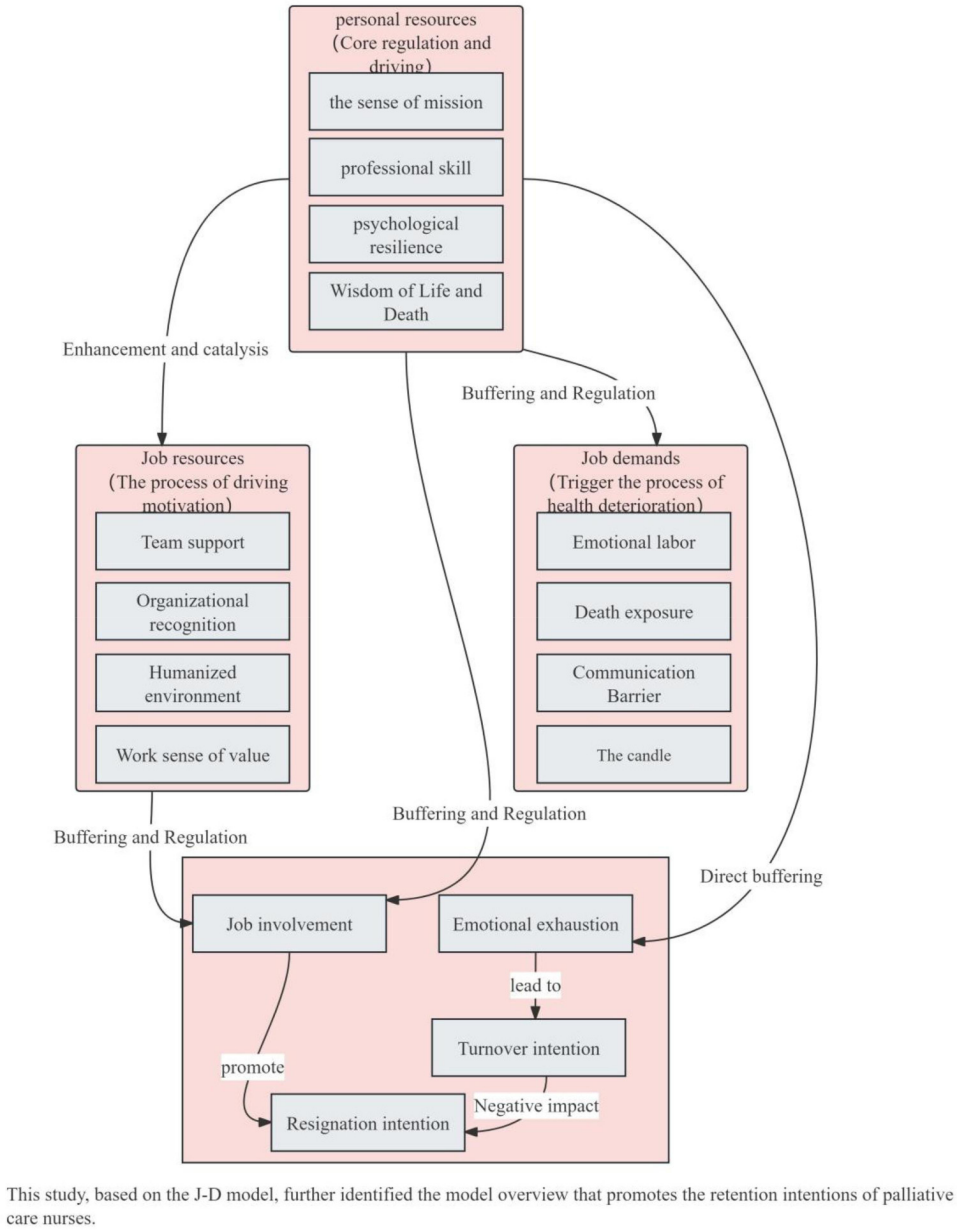
This stud, based on the J-D model, further identified the model overview that promotes the retention intentions of palliative care nurses.

The high-intensity demands of my work (emotional labor, exposure to death, communication difficulties, physical exhaustion) triggers emotional exhaustion and the intention to leave through the process of health deterioration. Meanwhile, abundant working resources (team support, organizational recognition, a humanized environment, and a sense of job value) stimulate work engagement through the motivational process and promote job retention. More importantly, the personal resources of nurses (such as a sense of mission, professional expertise, resilience, and wisdom in dealing with life and death) play a central role In this dynamic balance, they not only exert a direct motivating effect but also mitigate the negative impacts of work demands and amplify the positive effects of work resources.It can not only directly exert an incentive effect, but also buffer the negative impact of work demands and enhance the positive effect of work resources. Figure 2 presents an overview of the model discovered in this study, which is based on the J-D model and further elaborates on the facilitating factors for the retention intentions of hospice nurses.

### 4.1 The diversity and specificity of work requirements

Palliative care has developed relatively slowly in Europe and Asia. It began to be recognized by people gradually after 2000 (39). Hospital managers, palliative care teams, patients and their families, as well as the society, have insufficient understanding and knowledge of palliative care (40, 41).

Nurses in this role need to undertake multiple tasks, which requires them to possess a more comprehensive professional knowledge system. Among these tasks, “emotional labor” and “death exposure” represent core demands faced by nurses. The heavy workload and lack of recognition subject nurses to multiple physical and mental pressures.

### 4.2 Protection and incentive of work resources

Work resources seem to be a key factor for nurses’ health, showing a positive correlation with work demands and a negative correlation with burnout (42). Heavy workload, meager salary and insufficient staffing are the main factors that prompt nurses to consider leaving their jobs (43), Shortening the duty hours, reducing night shifts, providing more rest, and creating a safe and harmonious working environment are important reasons for nurses to consider staying in their positions (44, 45).

Research shows that clearly defining role divisions and providing team support can significantly reduce nurse burnout, with the emotional exhaustion level of nurses decreasing by 32% (46).

In addition, a reasonable nurse-patient ratio has a decisive impact on retention intention. When the nurse-patient ratio in palliative care wards is maintained below 1:3, nurses’ job satisfaction increases by 41% (47).

Those who work more than 8 h a day and care for over 10 terminally ill patients have a higher degree of job burnout (48), Spiritual care is at the core of palliative care. However, the spiritual care that patients hope to receive often does not match what medical staff intend to provide (49).

Nurses face obstacles such as not knowing how to break the ice when providing spiritual care, lacking relevant training, being busy with clinical work and having no time, and being confused about the differences between spirituality and religion (50, 51), Narrative medicine is not only a way for us to provide spiritual care, but also can alleviate the professional burnout of medical staff and heal themselves (52, 53). Social support, especially team cohesion, is a precious resource. This study also emphasizes the substantial support at the organizational level (such as salary and working environment), and points out that without these, it is difficult to sustain nurses’ enthusiasm and long-term commitment to their work merely by relying on team support.

### 4.3 Dynamic adjustment of personal resources

The topic of death is often rarely openly discussed because people consider it taboo and inauspicious (54). Therefore, many terminally ill patients are not aware of their condition. Family members insist on actively treating and rescuing the patients, and sometimes even ask nurses to conceal the fact of impending death from the patients. This practice, in itself, contradicts the purpose of palliative care.

Although the nurses providing palliative care are aware that such meaningless rescue and treatment will increase the patient's financial burden, cause them more physical and psychological pain, At the same time, the family members will also regret their current decision for not allowing the patient to have a peaceful death (55).

The nurses, however, are powerless in the face of such moral conflicts (56), Especially when it comes to the emotional exhaustion and moral distress caused by the deaths of children, it is even more pronounced (57).

In our research, we found that personal resources such as a sense of mission and professional skills offer nurses “reasons” for retention, serving as direct motivation. Moreover, psychological resilience and life wisdom endow them with “ability” (regulatory function), enabling them to interpret stressful events more positively and utilize work resources more effectively.

And psychological resilience indicates that in the field of palliative care, there exists a gain spiral between personal resources and work resources (58). Work practice has refined individual resources (such as enhancing professional skills and redefining the concepts of life and death). Enhanced individual resources empower nurses to better acquire and utilize work-related resources, such as actively seeking support and gaining more from training, thereby creating a positive cycle.

### 4.4 Practical insights and recommendations

Based on the aforementioned findings, we propose the following multi-level management and practical recommendations.

#### 4.4.1 Organization and management aspects

Managers should fully recognize the Acknowledge and publicly commend the unique job content and value of palliative care nurses, at the same time, actively organize the dissemination of life and death education among the general public to foster a correct view of life and death, which is conducive to alleviating the moral dilemmas faced by nurses. Properly allocate human resources, avoid overwork, Flexible rotation system, using the Maslach Burnout Inventory (MBI) (59) for assessment, to understand the situation of nurses’ burnout and formulate corresponding adjustment strategies to alleviate nurses’ burnout. Regularly organize ethical case discussions and death reflection meetings (60), to provide structured support for handling complex emotional and ethical issues. At the same time, enhance organizational resources, provide competitive salaries and benefits, and invest in the physical environment renovation of hospice care (such as setting up quiet corners and upgrading auxiliary facilities). Establish a clear career development path and a professional training system. When formulating the training plan for hospice nurses, it is necessary to pay full attention to training that is consistent with the cultural background (61). Especially for training on death and effective communication can help nurses have a correct understanding of death and reduce the anxiety brought by death (62). At the same time, training on resilience, mindfulness and self-compassion should be incorporated into the continuing education programs for employees, systematically helping nurses enhance their self-adjustment capabilities. At the same time, the (Survey of Perceived Organizational Support) assessment scale can be used to evaluate the nurses’ perception of the organizational support aspect (63).

#### 4.4.2 Team level

The team is the most direct and important social support system for palliative care nurses. Given the high regard nurses in this study have for team cohesion, we suggest establishing and formalizing regular multidisciplinary team meetings, such as weekly case discussions, to ensure full participation from all members, including doctors, nurses, social workers, psychologists, and volunteers. The meeting should not merely serve as a platform for transmitting medical information; rather, it should also serve as a platform for sharing emotional burdens, discussing ethical challenges, and jointly developing personalized care plans. Establish formal “peer support groups” and “mentoring systems” within the department, pairs comprising experienced senior nurses and junior nurses were established. Not only did they provide professional skills guidance, but they also paid attention to their psychological adaptation and emotional health.Encourage regular and formal communication, and provide new nurses provide a safe avenue for handling complex emotions and professional dilemmas. Regularly arrange team-based activities, including group mindfulness sessions and narrative reflection exercises. These activities are designed to assist team members in collectively addressing the sadness, stress, and setbacks encountered in their work, while also learning to maintain a healthy psychological boundary amidst deep empathy. Encourage the team to establish their own unique rituals, like conducting a brief internal farewell ceremony following a patient's death, regularly organizing team-building days, and jointly celebrating small successes at work. These rituals serve to affirm the significance of the work and reinforce team cohesion.

#### 4.4.3 Individual level

Ultimately, maintaining the mental health and retention intention of palliative care nurses hinges on enhancing and preserving their personal resources.Nevertheless, such individual-level intervention does not transfer the responsibility to the nurses,rather, it necessitates the organization to proactively provide resources and platforms and systematically cultivate nurses’ self-regulation ability and professional identity, such as incorporating resilience training and self-care training into the formal continuing education system, such as mindfulness stress reduction (64), emotion management (65), self-care and grief counseling etc. These trainings should not consist of sporadic lectures but rather be structured as comprehensive courses. Simultaneously, they should encourage nurses to engage in professional reflection through methods such as writing and sharing stories, this helps them integrate the challenges, values and confusions in their daily work, reframe the meaning of their work and achieve cognitive restructuring. Finally, reduce overtime work in non-emergency situations to ensure that nurses enjoy complete rest.

## 5 Limitations

This study has some limitations. Firstly, as a qualitative study, the samples were all from the same region and the same hospital. This limits the generalizability of the research results. The unique cultural and medical system characteristics of this institution may differ from those of other hospitals. Compared to the nurse-patient relationship in our background, the relationships in some Western or team-based models are more flattened, which may affect the perception and reporting of work demands and resources. Therefore, our research results should be used with caution when extending to other environments, and the similarities and differences of the situations should be taken into account. In the future, multi-center studies need to be conducted in different hospital types and cultural backgrounds to test the robustness and generalizability of the results. Secondly, the interviews mainly focused on the nurses’ own perceptions. In the future, perspectives from managers and patients’ family members can be incorporated for multi-source verification. This recruitment was issued by the head nurse. There might be some degree of selection or response bias when the nurses participated. Future studies can further reduce the bias by using a recruitment method that is completely anonymous, allows independent registration, and does not involve the managers of the departments.

Finally, this study has revealed the gain spiral between personal resources and work resources. This is a dynamic process, and in the future, a longitudinal tracking design may be employed to empirically examine the developmental trajectory of this spiral.

## 6 Conclusion

In conclusion, this study, by applying the JD-R model, reveals that the retention intention of palliative care nurses is not determined by a single factor. Rather, it is the result of a complex and dynamic balance among work demands, work resources and personal resources. To stabilize this precious team, it is far from enough to rely solely on the dedication and resilience of individual nurses. Medical institutions must adopt systematic and multi-level intervention strategies. While striving to optimize work demands, they should also consciously invest in and build supportive work resources, and empower the personal growth of nurses. Only in this way can the well-being of nurses be effectively promoted, and ultimately the high quality and sustainable development of palliative care services be guaranteed.

## Data Availability

The original contributions presented in the study are included in the article/Supplementary Material, further inquiries can be directed to the corresponding author.
